# The Management of Infectious Diseases in Comorbidity with Tuberculosis

**DOI:** 10.3390/medicina58101406

**Published:** 2022-10-07

**Authors:** Tafadzwa Dzinamarira, Mohd Imran, Claude Mambo Muvunyi

**Affiliations:** 1 ICAP, Columbia University, Harare P.O. Box 28, Zimbabwe; 2 School of Health Systems and Public Health, University of Pretoria, Pretoria 0002, South Africa; 3 Department of Pharmaceutical Chemistry, Faculty of Pharmacy, Northern Border University, Rafha 91911, Saudi Arabia; 4 College of Medicine and Health Sciences, University of Rwanda, Kigali 3286, Rwanda; 5 Rwanda Biomedical Centre, Kigali 7162, Rwanda

Tuberculosis (TB) infection is instigated by the bacillus *Mycobacterium tuberculosis* (MTB). Before COVID-19, TB was the main cause of infectious disease deaths worldwide [1]. MTB has infected about one-fourth of the global population. A six-month medication regimen can effectively treat 85% of those who contract TB [1]. The comorbidity between TB and other infectious diseases is very common in TB-endemic regions. Human immunodeficiency virus (HIV), malaria, toxoplasmosis, sexually transmitted infections, viral hepatitis, giardiasis, leishmaniasis, and pneumonia are a few examples of infectious diseases that can exist concurrently with TB [2]. The impact of co-infections and the management of TB patients who are also infected with HIV, malaria, toxoplasmosis, and hepatitis B will be covered in this article.

Between 1990 and 2010, sub-Saharan Africa’s TB notification rates increased five times due to the HIV epidemic. TB causes most HIV-related deaths worldwide, and HIV escalates the possibility of TB infection by 20–40 times. Up to 77% of African TB patients also have HIV [2]. TB infection also accelerates the advancement of HIV infection to AIDS, while HIV accelerates the progression of TB [3]. The toxicities and pharmacokinetic interactions between the medications used in TB treatment and antiretroviral therapy (ART) may make treating HIV and TB more difficult. The regimens may need to be changed to lessen side effects and non-adherence to treating both illnesses. HIV-infected TB patients who start using ART have higher survival and cure rates [4]. TB therapy among HIV-positive individuals (PLHIV) should be prioritized and initiated immediately after TB diagnosis. Prompt TB therapy stops TB transmission and lowers TB-related deaths. The length of TB treatment for PLHIV with TB should be the same as for PLHIV without HIV. ART should be started within the first eight weeks of initiating TB treatment. However, ART should start within two weeks for patients with CD4+ counts of 50 cells/mL or above. ART initiation should be postponed in cases of TB meningitis until after the continuous phase of TB treatment is finished [5].

Rifampicin, one of the drugs used in TB treatment, induces the cytochrome P450 liver enzyme system. This effect lessens serum concentration levels of non-nucleoside reverse transcriptase inhibitors (NNRTIs), protease inhibitors (PIs), and integrase inhibitors. Therefore, Integrase inhibitors and PIs must be given at higher doses to retain their efficacy. Rifabutin, which does not significantly stimulate the cytochrome P450 liver enzyme system, may be used as an alternative to rifampicin. Rifabutin’s dose should be cut in half if used with PIs because PIs raise serum levels of Rifabutin. Since the serum concentration of efavirenz is not considerably impacted by co-administration with rifampicin, an efavirenz-based regimen can be administered alongside the typical anti-TB medications rather than one that contains PIs [6]. Clarithromycin should also be avoided since it is a substrate and an inhibitor of cytochrome P3A in patients with multi-drug resistant TB who are on ART. Multiple medication interactions with NNRTIs and PIs will emerge from this. Additionally, due to the nephrotoxicity of both the anti-TB injectable drugs and tenofovir, the use of tenofovir-based regimens in conjunction with anti-TB injectable therapies should be undertaken with caution [3].

Immune reactions brought on by malaria reduce the host’s ability to fight against chronic TB infection. Anemia, cough, and respiratory distress are symptoms, clinical signs, and consequences common to TB and malaria. These similarities generally lead to a delay in diagnosis and the start of treatment. Due to the immunological interactions between malaria and TB, this delay may upsurge the mortality of TB patients [7]. Rifampicin induces the hepatic cytochrome P450 enzyme system, which lowers the serum quantities of artemisinin and its derivatives and, at the same time, makes treating TB and acute malaria more challenging. To employ the readily accessible artemisinin derivatives, patients on a rifampicin-containing regimen should have their rifampicin substituted with Rifabutin if they contract malaria [2].

Co-infections of TB and hepatitis B are common in nations with high TB prevalence. Hepatitis B infection may be able to reactivate and exacerbate TB symptoms, which can have negative clinical outcomes. Damaged liver from hepatitis B makes the liver more vulnerable to drug-induced harm. Treatment for TB may raise the risk of liver failure in those with liver impairment [8]. Poor patient outcomes and treatment responses are linked to concurrent hepatitis B and TB infection. Poor bioavailability caused by hepatitis infection-related frequent vomiting may be to blame for the poor treatment response to TB. If a patient with acute hepatitis B contracts TB, their TB treatment may need to be postponed. It has been demonstrated that treating hepatitis B with antiviral medications promptly after receiving a TB diagnosis reduces the risk of liver damage brought on by the hepatotoxic effects of anti-TB medications [9].

*Toxoplasma gondii* infection affects about one-third of the global population. MTB and *T. gondii* are common in developing countries. Although *T. gondii* does not cause major sickness in healthy persons, it does in patients with compromised immunity [10]. Th1 cytokine production decreases, and Th2 cytokine production increases during active TB infection. The reduction of cell-mediated immunity against toxoplasmosis from this alteration of the Th1 response toward the Th2 responses causes the reactivation of previous toxoplasmosis lesions or amplifies susceptibility to other infections. Additionally, toxoplasma co-infection worsens pulmonary TB by increasing its severity [11]. Rifampicin interacts with cotrimoxazole, a drug used to treat toxoplasmosis, creating treatment problems. The cytochrome P450 liver enzyme system is inhibited by cotrimoxazole, which causes rifampicin’s half-life to be longer and its adverse effects to be more severe. Therefore, it may be necessary to lower the dosage of rifampicin while treating toxoplasmosis in those taking it [12]. Because of the effects of the medications on the liver and their interactions, TB co-infection with other infectious diseases may complicate treatment options for both TB and the other infectious diseases, which may impair patient outcomes. Against this background, it is critical for researchers to share evidence on the inter-related pathophysiology, clinical manifestations, diagnosis, overlapping toxicity, and pharmacokinetic drug-drug interactions to inform health policies. In this Special Issue, we focus on manuscripts that present expert opinions and viewpoints, scoping and systematic review evidence, and primary research on the management of infectious diseases in comorbidity with TB.
